# Possibility of COVID-19 eradication with evolution of a new omicron variant

**DOI:** 10.1186/s40249-022-00951-7

**Published:** 2022-03-14

**Authors:** Moses Okpeku

**Affiliations:** Discipline of Genetics, School of Life Sciences, University of Kwazulu-Natal, Durban, South Africa

**Keywords:** COVID-19, Mutation, Omicron, Perspective

## Abstract

COVID-19 has been a global health concern since 2019 until date. Global concerted efforts to combat this pandemic has resulted in a number of vaccines distributed across the globe. Although the presence of these vaccines produced quick interventions, dynamic mutation in the causal virus and the continuous evolution of new stains that defy available vaccines has given rise to pertinent questions. The most recent emergence of a new COVID-19 virus variant (omicron), the rapid spread and overwhelming rate of morbidity and mortality has reopened these questions for debate. This commentary summarizes major view on these questions and concludes that multiple approach including social behavioural measures, vaccination and antiviral drug would speed up elimination process.

**Graphical Abstract:**

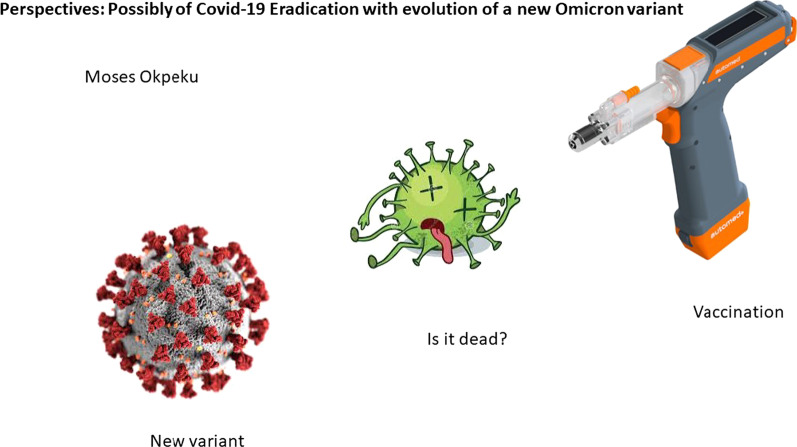

## Background

COVID-19, without warning suddenly appeared in 2019 and overwhelmed the world quickly [1]. Morbidity and mortality soared high, thousands died daily across the globe, a state of global emergency was declared and a global pandemic announced. Medical and non-medical interventions (lockdowns, controlled travels, use of facemask in public, social distancing) were put in place to limit the spread. Research and Development (R&D) companies raced at breakneck speed to find efficacious vaccines [2]. Quick production and fast distribution of vaccines, brought hope. However, with the evolution of novel, more fit and more deadly strains, hope became gleamed. The questions that came up on the media were; is eradication of COVID-19 possible? Like the Spanish flu, is it here to stay? etc. The emergence of the Omicron variant in South Africa recently and the rapid spread to other parts of the world further provokes these questions again. This article is a summary of perspectives in literature on the issue.

## Main text

**Fig. 1:**
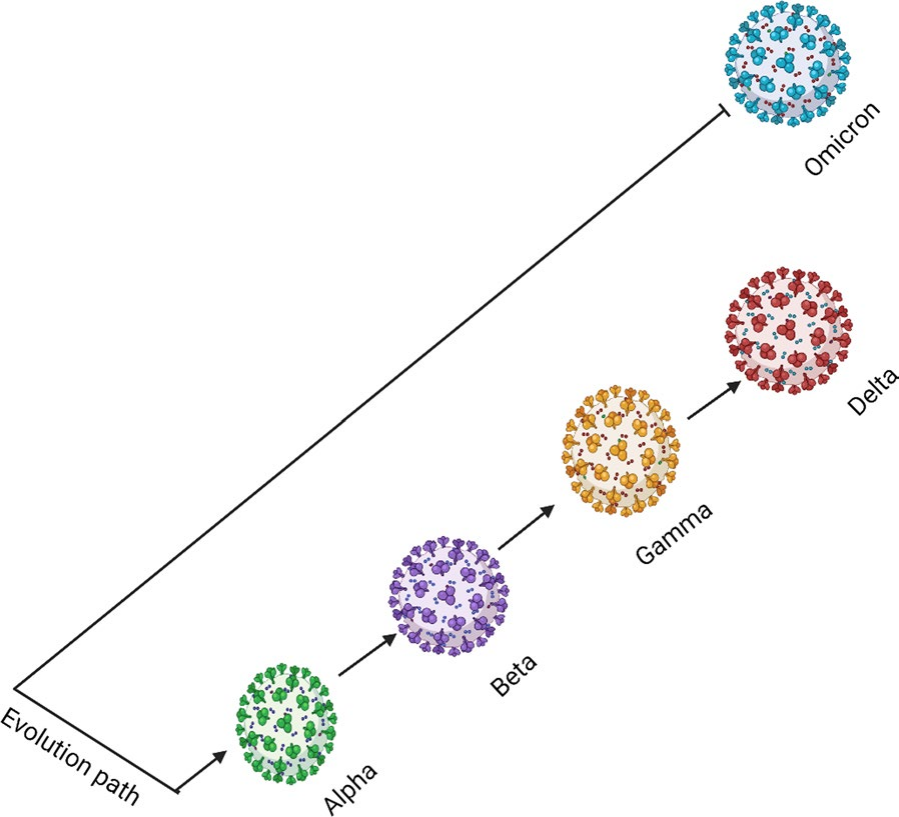
Parallel evolution of the omicron virus independent of other COVID-19 strains (created with Biorender.com)

The COVID-19 is a viral disease like the small pox and polio [3]. Immediate cure is not available but controlled with vaccines. Viruses have great potential to replicate rapidly; error induced during replications could potentially lead to a mutant virus type [4]. For mutation to occur, a lengthy resident in a host that allow gene flow between viruses is necessary. A mutant virus could possess properties that makes it more fit in aggravating disease condition. The COVID-19 since the first outbreak has evolved rapidly, novel and more deadly variants emerged from the old ones. However, the Omicron variant is thought to have evolved paralleled to earlier variants [5] (Fig. 1).

The first COVID-19 was first reported in China, Asia. The first variants from the original (Alpha variant) was first reported in United Kingdom (UK), in Europe was very effective in infection than the original as were subsequent variants; Beta (South Africa), Gamma (Brazil) and Delta (India). Etiology, dynamics of transmission, effectiveness of infection and viral load of the different variants did not follow the same route nor pattern; the common observation however was that newer variant were not necessarily more lethal or severe than their predecessor, but a progressive mutation in the spike protein enabling better transmission and infection was observed [5, 6]. The Omicron possess the most mutations in the spike protein, it is more advanced, and more effective at overwhelming acquired immunity [5, 8], even vaccinated persons, were confirmed infected, making Omicron superior in infection ability and casting doubts on the efficacy of available vaccines. To quickly arrest Omicron invasion, booster dosage of vaccines were recommended in some quarters [7]. However, booster dose for vaccines are usually produced post emergence of the mutant strain. Large scale data gathering, is needful also for determination of efficacy and setting dosage threshold, all of which is both costly and time consuming making this impractical in reality.

In South Africa, the dynamics of transmission of omicron compared with the Delta (the nearest predecessor) suggest it is less severe but more effective at by-passing the body defense systems [8]. Further increasing the possibility of a novel variant that defies available interventions. Endless cycles of mutant virus with superior infection ability, would make isolations and social distancing the panacea, but for how long? Lockdown cripples local and international economy, and reduce productivity [7, 8] at all levels. The implications and effects on socialization, commerce and global economy is not encouraging and hardly sought after now. In South Africa, the rate of infection and mortality is coming under controlled with vaccination and these preventive measures but where will the next mutant to continue the pandemic surface?

## Is COVID-19 eradication possible?

Is it here to stay and be managed like the flu? There are different perspectives to all of these questions and the debate is inconclusive. One perspective to it is that, eradication is possible, but those with this view are quick to admit that it would be very challenging and might take longer than expected [3]. Others argue that like the flu, the virus has come to stay [9]; but will require proper surveillance and great monitoring to keep it under control. While another school of thought believes that vaccination without pharmaceutical, intervention is the way. The search for a single efficacious vaccine must continue, yet it is difficult to predict and prepare vaccines awaiting a mutant that has yet to manifest. Proper sanitation and hygiene, spacing out and covering of the mouth and nose in public slow down the spread and may persist longer than anticipated. However, a multiple approach that combines all of these and the development and deployment of antiviral drugs for COVID should be encouraged and pursued. There is no known, vaccine for HIV aids but the global burden of the disease in now managed using antiviral drugs, as with other viral diseases like the influenza. Investment in antiviral drugs with multi-strain targets to support vaccination programs is proactive, especially when introduced timeously [10], will speed up elimination process. It would reduce viral load, interrupt incubation period, reduce mutations rate and evolution of difficult strains might be halted.

## Conclusions

COVID-19 eradication is not impossible, but an immediate solution seems not to be in view with the constant evolution of different variants of the virus. New variants might defile existing vaccines, research for improved vaccines to match new variants at the shortest time possible coupled with antiviral drugs is more proactive. To effectively identify and neutralize multi strains, bring down parasite load and reduce disease burden, it is recommended that research focused at drug design and implementation for fighting this minacious virus be intensified. No stone should be left unturned; this and other short time and longtime measures should be explored, with constant surveillance that incorporates rapid response.

## Data Availability

Not applicable.
